# The RESPITE trial: remifentanil intravenously administered patient-controlled analgesia (PCA) versus pethidine intramuscular injection for pain relief in labour: study protocol for a randomised controlled trial

**DOI:** 10.1186/s13063-016-1708-3

**Published:** 2016-12-12

**Authors:** Matthew Wilson, Christine MacArthur, Fang Gao Smith, Leanne Homer, Kelly Handley, Jane Daniels, On behalf of the RESPITE Collaborative Group, Linda Bradley, Seetharama Dinesh, Teresa Melody, Cody Allen, Karen Davies, Jennie Kerr, Philip Moore, Diane Whitehouse, Rebecca Chana, Deborah Horner, Aongola Ngenda, Jennifer Syson, Lesley Brittain, Kodaganallur Krishnan, Sarah Potter, Sarah Armstrong, Alexandra Oatley, Carol Snipe, Sandur Naresh, Sarah Davenport, Laurie Deeks, Sebastian Murray, Priya Krishnan, Maines Msiska, Jeremy Corfe, Elizabeth Turner, Elaine Johnson, Nuala Lucas, Jules Allt, Anna Fleming, Amanda Redford, Maria Okoisor, Julie Tebbutt, Jane Hillen, Angela Polanco, Megha Reddy, Zoe D’Souza, Anna Guy, Peter Rose, Holly Alcock, Samantha Roche, Suzanne Taylor

**Affiliations:** 1 Anaesthesia, School of Health and Related Research (ScHARR), University of Sheffield, 30 Regent St, Sheffield, South Yorkshire S1 4DA UK; 2 Maternal and Child Epidemiology, Public Health Building, Institute of Applied Health Research, College of Medical and Dental Sciences, University of Birmingham, Edgbaston, Birmingham, B15 2TT UK; 3 Anaesthesia, Critical Care and Pain, Perioperative, Critical Care and Trauma Trials Group, Institute of Inflammation and Ageing, Centre of Translational Inflammation Research, University of Birmingham Research Laboratories, Queen Elizabeth Hospital, Edgbaston, Birmingham, B15 2WB UK; 4 Birmingham Clinical Trials Unit (BCTU), Public Health Building, Institute of Applied Health Research, College of Medical and Dental Sciences, University of Birmingham, Edgbaston, Birmingham, B15 2TT UK

**Keywords:** Remifentanil, Pethidine, Epidural, Pain relief, Labour, Childbirth

## Abstract

**Background:**

The commonest opioid used for pain relief in labour is pethidine (meperidine); however, its effectiveness has long been challenged and the drug has known side effects including maternal sedation, nausea and potential transfer across the placenta to the foetus. Over a third of women receiving pethidine require an epidural due to inadequate pain relief. Epidural analgesia increases the risk of an instrumental vaginal delivery and its associated effects. Therefore, there is a clear need for a safe, effective, alternative analgesic to pethidine. Evidence suggests that remifentanil patient-controlled analgesia (PCA) reduces epidural conversion rates compared to pethidine; however, no trial has yet investigated this as a primary endpoint. We are, therefore, comparing pethidine intramuscular injection to remifentanil PCA in a randomised controlled trial.

**Methods/design:**

Women in established labour, requesting systemic opioid pain relief, will be randomised to either intravenously administered remifentanil PCA (intervention) or pethidine intramuscular injection (control) in an unblinded, 1:1 individual randomised trial.

Following informed consent, 400 women in established labour, who request systemic opioid pain relief, from NHS Trusts across England will undergo a minimised randomisation by a computer or automated telephone system to either pethidine or remifentanil. In order to balance the groups this minimisation is based on four parameters; parity (nulliparous versus multiparous), maternal age (<20, 20 < 30, 30 < 40, 40+ years), ethnicity (South Asian (Pakistani/Indian/Bangladeshi) versus Other) and induced versus spontaneous labour.

The effectiveness of pain relief provided by each technique will be recorded every 30 min after time zero, until epidural placement, delivery or transfer to theatre, quantified by Visual Analogue Scale. Incidence of maternal side effects including sedation, delivery mode, foetal distress requiring delivery, neonatal status at delivery and rate of initiation of breastfeeding within the first hour of birth will also be recorded.

Maternal satisfaction with her childbirth experience will be determined by a postpartum questionnaire prior to discharge from the delivery ward.

**Discussion:**

The RESPITE trial’s primary outcome is the proportion of women who have an epidural placed for pain relief in labour in each arm.

**Trial Registration:**

Current Controlled Trials registration number: ISRCTN29654603. Registered on 23 July 2013.

**Electronic supplementary material:**

The online version of this article (doi:10.1186/s13063-016-1708-3) contains supplementary material, which is available to authorized users.

## Background

*Why is there a need to determine epidural conversion rates for intervention with remifentanil patient-controlled analgesia or pethidine for pain relief in labour?*

Childbirth can be extremely painful and the provision of pain relief during labour is a humanitarian duty and a vital component of a positive maternal experience. The majority of women who deliver in modern obstetric units choose a pharmacological method of pain relief, including Entonox (50% N_2_O and 50% O_2_ mixture), the injection of opioids or neuraxial analgesia by epidural placement. The commonest opioid used in labour is pethidine, administered by intramuscular (im) injection [1]. However, the effectiveness of pain relief provided by pethidine has long been challenged [2]. Its shortcomings are more serious when set against known side effects including maternal sedation, nausea and potential transfer across the placenta to the foetus [3]. More than a third of women who receive pethidine subsequently require an epidural due to inadequate pain relief [4]. Epidurals provide highly effective pain relief, but increase the risk of a forceps or suction delivery [5–7]. Instrumental deliveries are associated with various long-term disadvantages including increased likelihood of faecal incontinence [8], sexual dysfunction [9, 10] and extended hospital stay. Modern ‘low-dose’ epidural regimens reduce this impact on labour but do not completely mitigate the effect.

Patient-controlled analgesia (PCA) comprises drug administration via an intravenous drip with a small dose given each time a woman presses a button, giving her control over her own pain relief. The pump is programmed to ensure that the maximum dose allowable is within the safe range in a given time period. This form of delivery of pain relief matches the drug dose to pain sensation within the relevant time frame, which is not possible using a single-dose im injection [11]. Whilst PCA is in widespread use for acute pain relief after surgery it has only a limited role in obstetrics.

Remifentanil is a novel synthetic opioid with a very rapid onset (blood-brain equilibration 1.2–1.4 min) and short duration of action (context specific half-life 2–3 min) [12], giving it an analgesic profile which potentially makes it effective for providing pain relief over one to two uterine contractions after a single intravenously administered dose. It is subject to rapid redistribution and metabolism by nonspecific blood and tissue esterases, negating the potential for accumulation in mother or foetus [13]. Administration of remifentanil by PCA has been investigated in several small studies in comparison to pethidine and been shown to provide useful, although not complete, pain relief in labour [14–16]. Thus far, there is no evidence of detrimental neonatal effects in comparison to other opioids [13, 15, 17].

Many UK units offer this form of pain relief in cases where pain relief is requested, but an epidural is contraindicated, for example, in the case of maternal blood clotting abnormality or platelet dysfunction. However, the use of remifentanil PCA is not currently widespread or routine [18]. Crucially, there is some evidence from the studies performed thus far that the proportion of women who require rescue pain relief with an epidural after remifentanil PCA is reduced in comparison to pethidine [13], although no study has yet investigated this as a primary endpoint. If such an effect were proven and remifentanil demonstrated to be at least as safe and effective as pethidine, the number of women requiring an epidural in labour could potentially be reduced with a concomitant beneficial reduction in instrumental vaginal delivery [5–7] and its associated morbidity.

Numerous clinical studies have examined the effectiveness of pain relief in labour provided by remifentanil PCA. These studies have resulted in the refinement of dose administration techniques to provide the optimum balance between effectiveness and maternal safety.

An additional table file (see Additional file 1: Table S1) summarises the pertinent remifentanil studies to date and gives details on study size, comparator and the rate of conversion to epidural analgesia, if reported. The heterogeneity of dosing regimen and the opioid techniques used for comparator are immediately apparent; however, a degree of consistency in epidural conversion rate in direction and proportion emerges. It is notable that the only study which reported a higher epidural conversion rate with remifentanil used a substantially smaller drug dose than those in more recent studies [16]. Thus, inadequate pain relief may have influenced maternal decisions to request neuraxial blockade.

Epidural conversion rates of approximately 10% (range 5% to 19%) are commonly reported after remifentanil PCA regimens using bolus doses of 40 μg or equivalent. This compares to conversion rates of greater than 30% (range 17% to 39%) being representative in women receiving pethidine. A systematic review [19] conducted in 2011, included two studies [4, 20] reporting epidural conversion rates for remifentanil as half that for pethidine, with two other studies [16, 21] showing no difference in progression to epidural.

## Methods

### Aim

The primary aim of the RESPITE trial is to determine whether remifentanil PCA, administered for pain relief in childbirth, reduces the requirement for progression to epidural analgesia relative to pethidine im injection. Secondary questions regarding the relative effectiveness of pain relief, maternal and neonatal indices of wellbeing and maternal satisfaction will be addressed.

### Trial design

RESPITE is a randomised, open-label, multicentre trial comparing intravenously administered remifentanil PCA with pethidine im injection in women in established labour requesting systemic opioid pain relief.

#### Primary outcome

The primary outcome is the proportion of women who have an epidural placed for pain relief in labour in each arm after randomisation. This does not include regional block (spinal or epidural) sited specifically for the purpose of emergency caesarean section.

#### Secondary outcomes

- The effectiveness of pain relief provided by each technique, quantified by Visual Analogue Scale taken every 30 min after time zero, until epidural placement, delivery or transfer to theatre
- The incidence of maternal side effects, up to the end of 3rd stage of labour, including:
  - ◦ Excessive sedation score (not rousable by voice, sedation score of ≥4)
  - ◦ Oxygen saturation (pulse oximetry) <94% whilst breathing room air
  - ◦ Nausea requiring antiemetic administration
  - ◦ Requirement and indication for supplemental oxygen
  - ◦ Respiratory depression (respiratory rate below eight breaths/min)
- Delivery mode (spontaneous vaginal, instrumental vaginal, caesarean section)
- Incidence of foetal distress requiring delivery
- Neonatal status at delivery:
  - ◦ Apgar score at 5 min
  - ◦ Incidence of foetal acidosis determined by umbilical cord gas analysis (if performed)
  - ◦ Requirement for neonatal resuscitation
  - ◦ Incidence of, and indication for, admission to neonatal care
- Rate of initiation of breastfeeding within the first hour of birth
- Maternal satisfaction with childbirth experience determined by postpartum questionnaire prior to discharge from the delivery ward

### Setting

Women will be recruited from multiple (fewer than 10) NHS trusts across England. Sites are able to participate in RESPITE if a care pathway is present which allows eligible women delivering at the site to be randomised between remifentanil and pethidine.

### Eligibility criteria

Women who are admitted to labour ward who fulfil all the following inclusion criteria and none of the exclusion criteria will be eligible to be randomised:

#### Inclusion criteria

- 16 years of age or older
- Beyond 37 + 0 weeks’ gestation
- In established labour, defined as regular painful contractions, irrespective of cervical dilatation, with vaginal birth intended
- Able to understand all information (written and oral) presented (using an interpreter if necessary) and provide signed consent
- Not participating in any other clinical trial of a medicinal product
- Live, singleton pregnancy with cephalic presentation
- Requesting systemic opioid analgesia

#### Exclusion criteria

- Contraindication to epidural analgesia
- Contraindication to im injection
- History of a previous adverse reaction to pethidine or remifentanil
- Patients taking any long-term opioid drug therapy including methadone
- Systemic opioid pain relief in the last 4 h administered by intravenous or intramuscular injection. (Medications administered per os comprising opioids alone or in combination preparations, administered in this 4-h period, are permitted)

#### Assessment of eligibility

Under the principles of Good Clinical Practice (GCP), the decision as to whether a patient is eligible for entry into a trial is considered to be a medical decision and, therefore, must be made by a medically qualified doctor. In the RESPITE study, this decision can be made by an anaesthetist or an obstetrician, but it is acknowledged that physicians are not routinely present when a woman requests opioid analgesia. The responsibility for eligibility assessment can be delegated to a research midwife or nurse and this decision will be made at a site level following local risk assessment in relation to the site’s organisation and practice, and should form part of the feasibility assessment for site approval. Eligibility for participation will be ratified by a GCP-trained physician.

### Randomisation

Randomisation will be carried out via a web-based central service (with staffed telephone back-up during office hours) based at Birmingham Clinical Trials Unit (BCTU). A 24-h, 7-day, interactive, telephone-based randomisation service will also be provided by the Health Services Research Unit at the University of Aberdeen. The web-based central service is only available to those named on the delegation log, whereas both telephone services are also available to clinical midwives, nurses and operating department practitioners (ODPs).

To obtain a randomised allocation and trial number, the person performing the randomisation process will need to verify a woman’s date of birth, confirm that all eligibility criteria have been met and provide the minimisation variables. At randomisation a confirmatory email will be sent to the randomising investigator, the local principal investigator (PI) and the research midwife/nurse. A ‘minimisation’ procedure using a computer-based algorithm will be used to avoid chance imbalances in parity, an important prognostic variable, which will be considered as an ordinal variable.

Minimisation variables will be:

1. Parity: nulliparous versus multiparous
2. Maternal age: <20, 20 < 30, 30 < 40, 40+ years.
3. Ethnicity: South Asian (Pakistani/Indian/Bangladeshi) versus Other
4. Induced versus spontaneous labour

The procedures for randomisation will be fully documented and tested prior to the start of the trial and monitored by BCTU.

### Pain relief

Women will be randomly allocated in a 1: 1 ratio to either:

1. Remifentanil PCA (intervention group) or
2. Intramuscular injection of pethidine (control group)

#### Remifentanil

A dedicated intravenous cannula for remifentanil administration will be inserted into the woman ’s hand or forearm. The PCA pump will be preprogrammed by anaesthetic staff with a regimen to provide a bolus of 40 μg remifentanil with a lockout interval of 2 min. This dose regimen is based on sample guidelines adapted from those used in the introduction of remifentanil PCA into clinical practice in some UK labour wards [22] and reflects those used in the largest study up to 2010–2011 [20, 23]. In the event of excess sedation being recorded by regular observation of respiratory function, the regimen will be altered by reduction of the remifentanil bolus dose to 30 μg with a lockout interval of 2 min.

#### Pethidine

Pethidine will be delivered at a dose of 100 mg by im injection, up to 4-hourly in frequency, up to a maximum of four doses, making the maximum dose 400 mg in 24 h.

#### Intrapartum care

After the administration of analgesia, women will receive one-to-one midwifery care independent of group allocation. Clinical observations will be recorded every 30 min for both monitoring of the women’s status and for trial purposes, including respiratory rate, continuous oxygen saturation monitoring by pulse oximetry, numerical sedation score (on a scale of alert to unresponsive) and pain, using a Visual Analogue Scale pain score of 0 to 100.

The time at which active labour (regular painful contractions and more than 3-cm cervical dilatation) and second stage (dilatation of at least 10 cm) is diagnosed by vaginal examination, will be recorded but the degree of dilatation is not relevant to determination of eligibility.

Indications for contacting a physician anaesthetist are excessive sedation (not rousable by voice), a respiratory rate below eight breaths/min or oxygen saturation <94% despite supplemental inspired oxygen therapy

Women are free to request epidural pain relief at any point after trial entry. Neither the consenting physician, research midwife/nurse nor ODP will advise, offer opinion or be involved with a decision to proceed to epidural. The decision will be taken by a woman participating in the study supported by attending clinical (nonresearch) midwifery staff responsible for their care, if required. A maternal request for epidural analgesia will be treated according to local labour ward practice including assessment by attending midwifery and anaesthetic staff. The precise details of epidural technique will be dictated by local labour ward protocols. In the rare instance of epidural placement being recommended for medical indications arising during labour, this will be recorded. There is no reason to expect an imbalance of such an event between allocated groups.

Once effective epidural pain relief is established, the administration of opioid drugs will be discontinued irrespective of trial group allocation. Maternal observations will be recorded up to the point of epidural placement, delivery or transfer to operating theatre. Neonatal status will be recorded at delivery.

### Follow-up

The study is primarily confined to the time period when a woman is in childbirth. Women enter the study from the time that they request opioid pain relief in labour and remain in the study for the duration of labour until both mother and baby have been discharged from hospital.

The period is, therefore, measured in days. Active data collection will only occur up to the point of child birth. There will be a single maternal contact thereafter to administer a brief questionnaire which includes metrics of maternal satisfaction. Mean stay in hospital after spontaneous vaginal delivery is 1–2 days. This period can be extended if delivery by emergency caesarean section has been required.

The trial schema which includes eligibility criteria and outcome measures is shown in Additional file 2: Figure S1.

#### Monitoring of all types of adverse event

All types of adverse event which occur to a woman in the RESPITE trial will be reported as soon as possible after research staff become aware of the event. A Serious Adverse Event Form should be completed as fully as possible and sent via fax or email (from an NHS account) to the Trials Office. This form will include an assessment of causality. Upon receipt, the Trials Office will log details of the adverse event on that patient’s record on the central trials database and forward an anonymised copy of the form to the chief investigator.

Anonymised details of all adverse events will be presented to the Data Monitoring Committee (DMC) for scrutiny each time they meet. These anonymised details will be reported to the Research Ethics Committee (REC) and Medicines and Healthcare Products Regulatory Authority (MHRA) annually.

### Statistical analysis

#### Sample size

Epidural conversion rates of approximately 10% (range 5% to 19%) are commonly reported after remifentanil. This compares to conversion rates of greater than 30% (range 17% to 39%) being representative in women receiving pethidine. Taking a deliberately conservative estimate of intervention effect using these data, a reduction in epidural conversion from 30% (pethidine) to 15% (remifentanil PCA) is reasonable. To detect such a reduction with 90% power at *p* = 0.05, would require 161 women in each arm of the trial, yielding a sample size of 322 in total. Adjustment must also be made to account for attrition of the study population as labour progresses. A proportion of the women will require urgent delivery, by caesarean section, for foetal indications before a request for further analgesia can be made. Assuming that this will not represent more than 25% of the sample after randomisation (reflecting the national caesarean section rate) and a 15% emergency caesarean rate is more realistic, a further 48 women will be recruited to offset this attrition of participants who are not capable of reaching the primary outcome. Allowing for a modest unavailability of primary outcome data and nonadherence of 6%, a total sample size of 400 is, therefore, proposed.

A detailed analysis plan will be developed and agreed by the TSC and the DMEC. Demographic factors and clinical characteristics will be summarised with counts (percentages) for categorical variables, mean (standard deviation) for normally distributed continuous variables, or median (interquartile or entire range) for other continuous variables.

#### Primary analysis

The primary analysis will be a comparison of the management policies assigned at randomisation (intention-to-treat population.) The risk of the primary outcome in the remifentanil PCA group will be compared with the group and tested for significance at the two-sided 5% level of significance. In addition to the primary unadjusted analysis, a log-binomial model will be used to account for the minimisation variables. Risk ratios and 95% confidence intervals will be estimated. As the remifentanil PCA will generally only be available within the context of the RESPITE trial, we do not anticipate many protocol deviations; however, a limited incidence of nonadherence is predicted. The most likely cause is rapidly progressing labour with delivery occurring after randomisation, before allocated pain relief can be administered.

#### Subgroup analysis

Perception of pain during labour may be influenced by previous experiences, in particular previous labours, and this may, in turn, influence the request for epidural. Maternal parity will, therefore, be prespecified for subgroup analysis of primary outcome.

#### Handling missing data

The extent of missing data for the primary outcome should be limited, as it is routinely recorded in maternity records and can be collected retrospectively. The Maternal Outcome Satisfaction Questionnaire must be completed before discharge and research nurses/midwives should make every effort to collect this. Where these time-critical data are missing, multiple imputation methods may be considered to inform a sensitivity analysis.

### Data management and quality assurance

#### Data form confidentiality

Personal data and sensitive information required for the RESPITE trial will be collected directly from trial participants and hospital notes on paper Data Collection Forms, coded with the woman’s unique trial number and initials. Women will be informed about the transfer of this information to the RESPITE trial office at BCTU and asked for their consent. All personal information received in paper format for the trial will be held securely and treated as strictly confidential according to BCTU policies. All staff involved in the RESPITE trial share the same duty of care to prevent unauthorised disclosure of personal information. No data that could be used to identify an individual will be published.

#### Confidentiality of RESPITE database

RESPITE data will be electronically stored and managed on a dedicated, secure, encrypted trial database, specifically constructed for the purpose. The data will be entered onto a secure computer database, either directly via the Internet using secure socket layer encryption technology or indirectly from paper by BCTU staff. The RESPITE database at BCTU is managed under the provisions of the Data Protection Act and/or applicable laws and regulations.

Randomisation can be achieved either via the web-based randomisation module of the trial database, or by use of a touch-tone activated telephone system, via the University of Aberdeen, which synchronises with the RESPITE database.

Access to the randomisation module and data entry screens of the database will be controlled via individual username and passwords assigned to research midwives/nurses and physicians identified on the delegation log as having responsibility for data collection, and by BCTU trial staff. Transcription of paper forms on to the RESPITE database will be performed either at the site, or the paper form will be faxed to BCTU.

#### In-house data quality assurance and validation

The study will adopt a centralised approach to monitoring data quality and compliance. A computer database will be constructed specifically for the study data and will include range and logic checks to prevent erroneous data entry. Independent checking of data entry of paper questionnaires will be periodically undertaken on small subsamples. The trial statistician will regularly check the balance of allocations by the minimisation variables. Source data verification will only be employed if there is reason to believe that data quality has been compromised, and then only in a subset of practices.

#### Long-term storage of data

In line with the impending revision of the Medicines for Human Use (Clinical Trials) Regulations, once data collection is complete on all participants, all data will be stored for at least 25 years. This will allow adequate time for review and reappraisal, and in particular with the RESPITE trial, form the basis for further follow-up research. Any queries or concerns about the data, conduct or conclusions of the trial can also be resolved in this time. Limited data on the participants and records of any adverse events may be kept for longer if recommended by an independent advisory board.

Trial data will be stored within BCTU under controlled conditions for at least 3 years after closure. Long-term offsite data-archiving facilities will be considered for storage after this time. BCTU has standard processes for both hard copy and computer database legacy archiving.

## Discussion

This protocol describes the RESPITE randomised controlled trial, which will evaluate the effectiveness of remifentanil intravenously administered PCA compared with pethidine im injection (the current, standard opioid analgesic for women in established labour). This will be assessed by comparing epidural conversion rates in each study arm. This multicentre study will recruit 400 women in childbirth and the results will be used to make recommendations on the subsequent adoption of remifentanil into clinical practice in childbirth via publications and clinical guidelines.

Many UK units now routinely offer remifentanil PCA in cases where an epidural is contraindicated and a very restricted number make it available ‘on demand’ as an alternative analgesic intervention in labour. Those units which have pursued adoption into routine practice have now accumulated the experience of the safe administration of remifentanil PCA to several thousand women in childbirth. However, the summary of product characteristics for remifentanil states that there are insufficient data to recommend its use in labour or caesarean section, so in effect this usage is off label. The challenges of conducting clinical trials of investigational medicinal products (CTIMPs) to the standard of GCP required by regulatory authorities are well-known [24].

For RESPITE, the use of remifentanil outside its licenced indication places the trial in a higher-risk category according to the guidance of the MHRA [25]. This does not necessarily reflect an increased risk to the trial participants but an acknowledged lack of data. However, it increases the burden of risk management and monitoring on those conducting the trial.

Another challenge for RESPITE is the intrapartum recruitment of women in active labour. We have adopted the recommendation of the RCOG, in which the timing and extent of the information provided to women is adapted according to the risk of becoming eligible for the trial [26]. A considerable proportion of women require analgesia, so we ensured that all women booked to deliver at participating hospitals receive information about the study at the antenatal clinic, during their pregnancy and again on admission in labour. The majority of the eligibility criteria can be determined on admission in labour. To avoid a situation where consent is sought from a woman in pain and distress, consent to participate can be sought from the point that labour is considered to be established, or is induced, up to and including the point when the final eligibility criteria, that of requesting opioid analgesia, is apparent. This allows the research midwife to fully discuss the trial and gain valid consent from a woman who is considering participation, and also prepare the eligibility checklist to enable a delivery suite midwife to complete the final step of randomisation if, and when, the request for opioids is made by the woman. Randomisation may, therefore, continue even if the research midwife is not present, essential in intrapartum situations. It is widely accepted that suitably trained research nurses and midwives are appropriate persons to seek consent for participation in a clinical trial [27]. Under the principles of GCP, the decision as to whether a patient is eligible for entry into a trial is considered to be a medical decision and, therefore, must be made by a medically qualified doctor. In the RESPITE study, this decision can be made by an anaesthetist or obstetrician, but it is acknowledged that physicians are not routinely present when a woman requests opioid analgesia. Indeed, pethidine, although a controlled drug in UK law, can be independently prescribed by a qualified midwife. MHRA acknowledges that there are certain situations where the assessment of eligibility can be broadened to include professionals who are not medically qualified, such as midwives or nurses. This provision is subject to the following criteria:

- The role of nonmedical professionals in determining eligibility has been risk-assessed
- The role of nonmedical professionals is consistent with standard clinical practice and that arrangements for the clinical trial should not fall below that standard
- The duties involved in determining eligibility have been delegated by an appropriately trained and qualified member of site staff
- The eligibility assessment process should be overseen by a medically qualified doctor

It is imperative that the roles and responsibilities are clarified and communicated with the regulatory authorities prior to the start of any trial facing similar challenges. This exemption is essential for intrapartum research and other situations where nonmedical health care professionals with advanced prescribing responsibilities are the primary care providers.

### Trial status

RESPITE is currently recruiting.

## Additional files

Additional file 1: Table S1. Previous studies of remifentanil for analgesia in labour [20, 21, 23, 28]. (XLS 27 kb)
Additional file 2: Figure S1. RESPITE trial schema. (DOC 344 kb)

## Abbreviations

BCTU: Birmingham Clinical Trials Unit
CTIMP: Clinical Trial of an Investigational Medicinal Product
DMC: Data Monitoring Committee
DMEC: Date Monitoring and Ethics Committee
GCP: Good Clinical Practice
IM: Intramuscular
ISRCTN: International Standard Randomised Controlled Trial Number
MHRA: Medicines and Healthcare Products Regulatory Authority
NHS: National Health Service
NIHR: National Institute for Health Research
NRES: National Research Ethics Service
ODP: Operating department practitioner
PCA: Patient-controlled analgesia
PI: Principal investigator
PPI: Patient and Public Involvement
PRIME: Public and Researchers Involvement in Maternity and Early pregnancy group
RCOG: Royal College of Obstetricians and Gynaecologists
REC: Research Ethics Committee
ScHARR: School of Health and Related Research
TMG: Trial Management Group
TSC: Trial Steering Committee
