# Supplementary material for: The RESPITE trial: remifentanil intravenously administered patient-controlled analgesia (PCA) versus pethidine intramuscular injection for pain relief in labour: study protocol for a randomised controlled trial
Source: Trials. 2016 Dec 12;17:591. doi: 10.1186/s13063-016-1708-3 (PMC5153689; doi:10.1186/s13063-016-1708-3)
Supplement: Additional file 2: Figure S1. — RESPITE trial schema. (DOC 344 kb) [file 13063_2016_1708_MOESM2_ESM.doc]

RESPITE trial schema

**FLOW DIAGRAM**


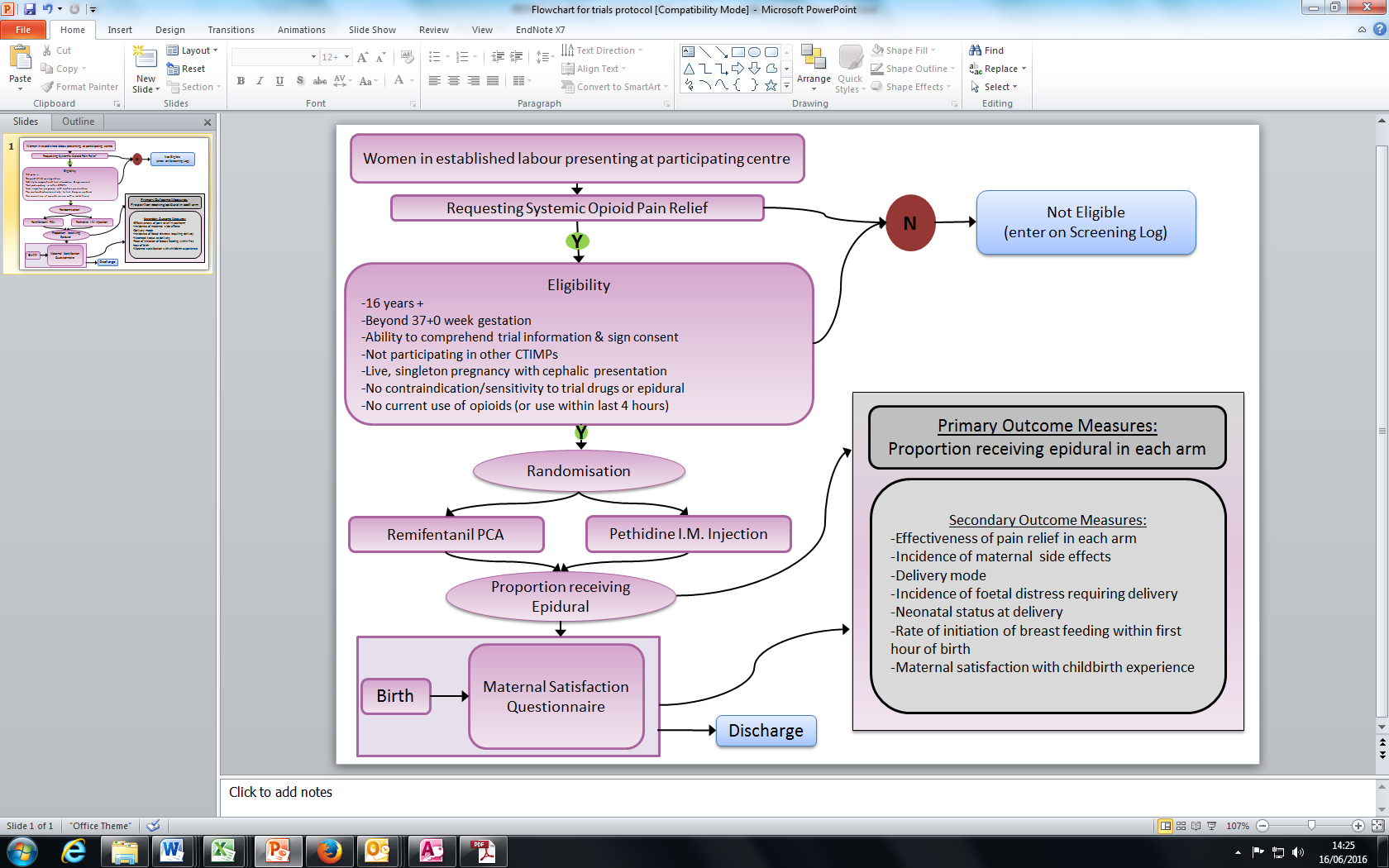


**Figure S1 RESPITE trial schema**. CTIMP, clinical trial of an investigational medicinal product; PCA, patient controlled analgesia; IM, intramuscular
